# The effects of interventions to integrate long‐acting reversible contraception with treatment for incomplete abortion: Results of a 6‐year interrupted time series analysis in hospitals in mainland Tanzania and Zanzibar

**DOI:** 10.1002/ijgo.14203

**Published:** 2022-04-21

**Authors:** Colin Baynes, Kathryn A. O’Connell, Grace Lusiola, Danielle Garfinkel, Justin Kahwa

**Affiliations:** ^1^ EngenderHealth Washington District of Columbia USA; ^2^ EngenderHealth Dar es Salaam Tanzania

**Keywords:** family planning, long‐acting reversible contraception, Postabortion care, Tanzania

## Abstract

**Objective:**

To evaluate an intervention that aimed at strengthening voluntary access to long‐acting reversible contraception (LARC) within postabortion care (PAC) in hospitals in mainland Tanzania and Zanzibar.

**Methods:**

From 2016 to 2018, we conducted PAC quality improvement interventions, emphasizing family planning (FP) counseling and voluntary access to LARC. Researchers conducted an interrupted time‐series analysis of service statistics compiled from 2014 to 2020 using segmented linear mixed effects regression models to assess the interventions' effect on postabortion contraceptive uptake.

**Results:**

The intervention in mainland Tanzania was associated with an immediate 38% increase in postabortion LARC uptake, a trend that declined from late 2016 to mid‐2020 to 34%. In Zanzibar, the intervention was associated with a gradual increase in LARC uptake that peaked in late 2018 at 23% and stabilized at approximately 15% by mid‐2020. Whereas the interventions in mainland facilities did not generate significant changes in postabortion FP uptake overall, the launch of interventions in Zanzibar in mid‐2016 was associated with a precipitous rise in that outcome over time, which plateaued at approximately 54% by 2019.

**Conclusion:**

Increased voluntary uptake of postabortion contraception was associated with the introduction of training in PAC, including FP, and quality improvement interventions and gains were sustained over time.

## Synopsis

EngenderHealth implemented an intervention to improve integration of treatment for postabortion complications and family planning, which achieved immediate effects that were sustained over time.

## 1 INTRODUCTION

Unintended pregnancies and subsequent abortions are problems worldwide and a driver of maternal mortality and morbidity, particularly in countries such as Tanzania, where there are social, cultural, and legal constraints to accessing comprehensive sexual and reproductive health services. Tanzania has one of the world’s highest maternal mortality ratios (410 per 100 000 live births) and a ratio of 21 abortions per 100 live births.^1^, ^2^ Accordingly, its government has expanded its program on postabortion care (PAC), which helps save the lives of women suffering from abortion complications by providing emergency treatment and offering family planning (FP) counseling, including voluntary access to contraception, in the same visit. Most women requiring PAC in Tanzania are treated at the tertiary level, despite recent efforts aimed at decentralizing the service from district and regional hospitals to primary care settings.^3^ Decades of research have demonstrated the effectiveness of strengthening the FP component within PAC on immediate voluntary uptake of contraception and enhanced prospects for better health throughout patients’ lives.^4^ Indeed, women are eligible to use nearly all modern contraceptive methods immediately after PAC. This includes long‐acting reversible contraception (LARC), i.e. implants and intrauterine devices (IUDs), which can be inserted after PAC and once medical professionals have confirmed that the uterus is empty.^5^, ^6^ Leading global health agencies and professional associations have recognized the accumulation of evidence on postabortion FP as a high‐impact, evidence‐based intervention, and advocate for its large‐scale uptake throughout health systems worldwide.^7^

In response to these calls, numerous development partners have supported to low and middle income countries to introduce and scale up postabortion FP. This has resulted in the demonstration of valuable technical approaches and noteworthy improvements in the coverage, quality, and utilization of PAC and women’s postabortion uptake of contraception.^4^, ^8^ From 2016 to 2020, EngenderHealth, a global sexual and reproductive health and rights non‐governmental organization, carried out such an intervention in hospitals in mainland Tanzania and Zanzibar.^3^ This article reports on the outcomes of an interrupted time‐series analysis of the effects of this intervention on postabortion FP uptake comparing periods before, during, and after (i.e. pre‐intervention, early‐intervention, and sustainment periods) the EngenderHealth’s program.

## 2 MATERIALS AND METHODS

### 2.1 PAC and the Tanzanian health system

In 2014, EngenderHealth started the global *Postabortion Care Family Planning Project (PAC‐FP)* with funding from the United States Agency for International Development (USAID). In 2015, with support from USAID/Tanzania, *PAC‐FP* partnered with the Ministries of Health of Tanzania and the semi‐autonomous island region of Zanzibar and planned a phased program of technical assistance to local health systems in Geita and Mwanza regions on the mainland and Pemba and Unguja Islands of Zanzibar.

Even though the two regions operate under different Ministries and have received different levels of support to develop PAC services over time, PAC is configured relatively similarly in their health systems. Mainland Tanzania has received long‐standing external support to scale up PAC, starting in the early 2000s, and since then has established services in over 200 facilities in the Lake Zone, which includes Mwanza and Geita. In these regions, the health system has a typical three‐level pyramid structure with tertiary and regional and district facilities, intermediate health centers, and, closest to communities, primary healthcare centers. Before the project, hospitals provided the widest range of PAC care including vacuum aspiration for routine incomplete abortion and advanced surgical care for severe complications (e.g. laparotomy), and a wide range of FP methods were available in separate hospital settings. Health centers were able to provide treatment for routine complications of abortion and short‐term contraceptive methods. In health centers, also, FP services were provided separately. At lower levels of care, PAC services during this period experienced lapses in sustainment owing to intermittent patient flows and staff turnover and logistical challenges. According to policy, only surgical treatment, not misoprostol, could be used for treating abortion complications. In Zanzibar, which has received appreciably less external support to organize a regional PAC program, the vast majority of PAC services were provided at the Regional Referral Hospital in Unguja, with far fewer cases recorded per month throughout the network of district hospitals on both islands. Even though both types of facilities could treat abortion complications and provide postabortion contraception, the regional referral facility, Mnazi Moja Hospital, received most cases with severe complications. FP services in both settings were offered separately from PAC and limited to short‐acting methods. In Zanzibar, cases of routine incomplete abortion during this time were occasionally recorded at Primary Healthcare Units. At variance with the mainland PAC program, misoprostol has been recognized as an appropriate treatment for abortion complications in Zanzibar for some time, even though there were no official guidelines on this until *PAC‐FP* helped to develop them in 2018.

### 2.2 The intervention

*PAC‐FP* employed an implementation strategy that prioritized organizational development, capacity building, data‐use for decision making, and cascaded trainings. The approach blended didactic and experiential learning of the clinical and interpersonal competencies necessary to effectively treat abortion complications and help patients to meet their longer‐term reproductive health needs. The project team implemented two phases. During phase one (2016–2018), *PAC‐FP* directly supported Council Health Management Teams and facility staff in capacity building efforts focused on training, quality improvement (QI), and service integration. During phase two (2018–2020), the program supported the efforts of council health management teams to sustain the gains achieved in phase 1 and continue scale‐up.

**FIGURE 1:**
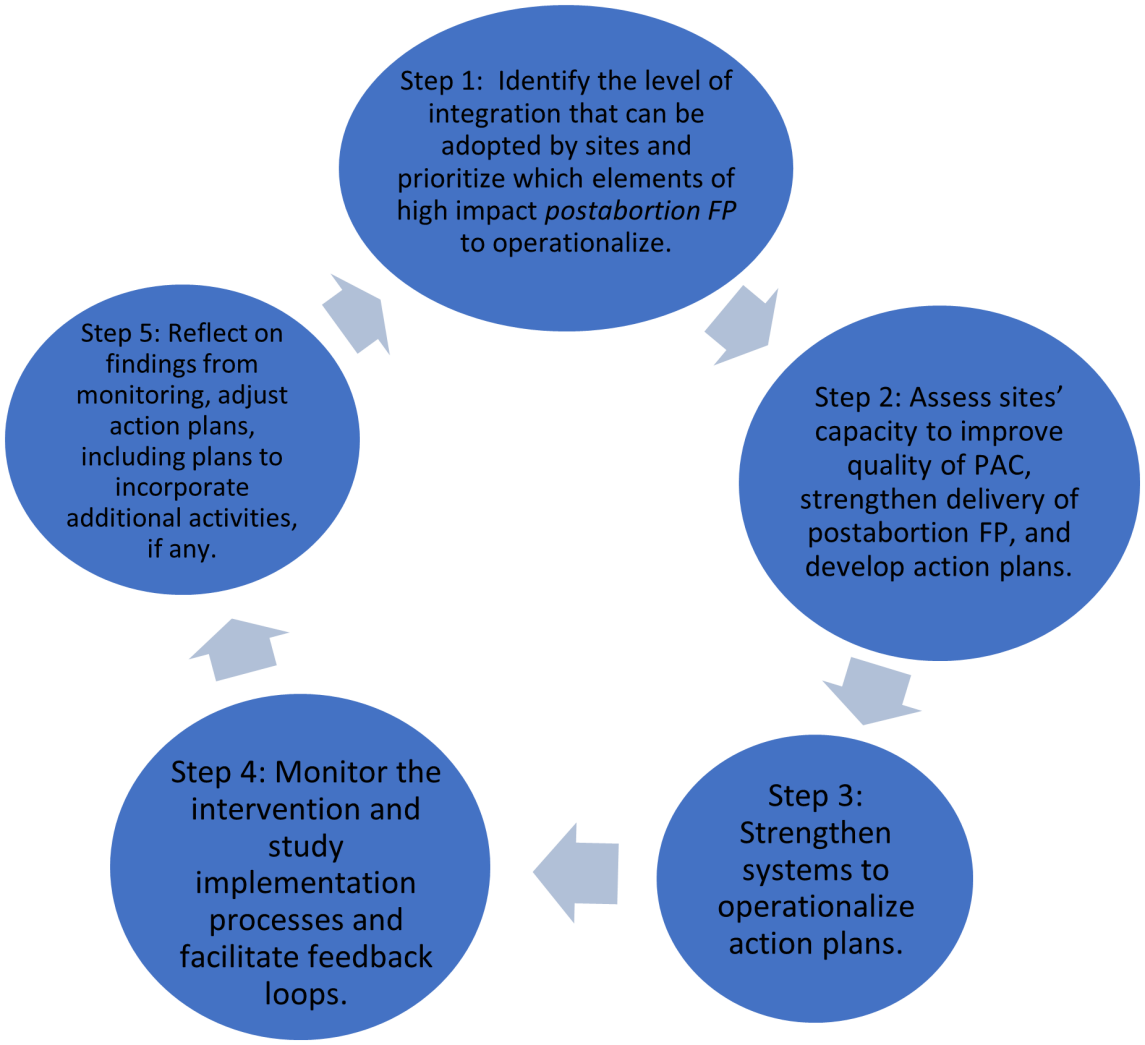
Five‐Step Framework for QI and Service Integration

The intervention used an iterative five‐step approach to embed the processes in district‐level healthcare management structures, hospitals, and points of care where PAC is available. This is illustrated in Figure 1.

During step 1, *PAC‐FP* worked with district‐level counterparts to conduct organizational capacity assessments (OCAs). OCAs illuminated district‐level barriers and facilitators to achieving project goals and established stakeholder consensus on next steps. In mainland Tanzania, where the PAC program was more mature and uptake of short‐acting methods of contraception was already high, stakeholders prioritized integrating LARC into the postabortion method mix.^9^ Whereas in Zanzibar, whose PAC program had received little external assistance, stakeholders chose to strengthen FP integration more gradually, focusing first on better counseling and availability of FP services, and the development of clinical guidelines on PAC that promote access to all types of postabortion contraception.^10^ These guidelines also give instruction on the strengthening of PAC, including postabortion FP, in primary care facilities, which had not been emphasized in prior work to help develop the Zanzibar PAC program. In both settings, OCA groups adopted a phased implementation approach that started in hospitals in 2016.

In step 2, OCA groups disseminated the results at selected hospitals and, in turn, facilitated facility‐level capacity assessments with the support of *PAC‐FP*. With this, hospitals' existing QI teams developed site‐specific action plans. In mainland Tanzania, this included updates to product placement and requisition, and integration of existing PAC and LARC trainings into modules and tools. In Zanzibar, QI teams also focused on FP product placement and requisition reforms, and established dedicated private settings in each hospital for PAC provision. Stakeholders in Zanzibar opted to adapt and use the centralized PAC and LARC training curriculum from the mainland as an initial step in developing region‐specific clinical PAC guidelines.

During steps 3 and 4, the QI teams implemented their action plans, receiving catalytic financial, material, and technical support from *PAC‐FP*, including PAC and LARC training. *PAC‐FP* monitoring and evaluation staff routinely compiled data on action plan achievement, the contextual factors affecting performance, and changes made to action plans as a result. *PAC‐FP* convened OCA and QI teams for periodic meetings in each region where teams shared, interpreted, and used results to inform efforts to accelerate progress and solve problems.

In step 5, OCA teams reflected on lessons and considered matters related to extending the intervention to primary care settings, engaging communities, and other strategic issues.

The project completed all steps by October 2018.

### 2.3 The evaluation

This study reports on the effect of the above cycle of implementation in eight hospitals in mainland Tanzania and six hospitals in Zanzibar. The analysis presented herewith is confined to this subset of the 65 health facilities that received support from *PAC‐FP*. They were the first to enroll in the project because the implementation strategy sought first to strengthen services and QI teams in tertiary sites, and thereafter, starting at step 5, utilize these facilities as “hubs” from which to cascade PAC‐related trainings and capacity building to improve PAC quality in primary care facilities in their respective catchment areas. Given that the objective of this study was to assess the long‐term impact of a postabortion FP QI intervention—particularly those changes that transpire over time as facility teams face challenges of sustaining early‐stage gains of an intervention—it was necessary to limit the study to those facilities that enrolled in the project at the earliest stage and were, therefore, observed for a sufficiently long period of time. Researchers conducted separate analyses for each region. The evaluation used data from PAC registers at respective hospitals. Researchers formulated an interrupted time‐series dataset with aggregated counts of the number of PAC patients, whether they received FP services, and contraception by method type accepted by clients per month for each hospital. Analysts delimited the dataset into three time‐segments: (1) January 2014 to July 2016, representing the pre‐intervention phase running through the end of step 2; (2) August 2016 to October 2018, the period starting with step 3 and ending in step 5; and (3) November 2018 to June 2020, a sustainment period, during which *PAC‐FP* continued to work with district partners, periodically making supervision visits to hospitals while focusing on supporting PAC improvements in lower‐level facilities. Researchers used the interrupted time series to conduct segmented regression analyses that examined two parameters for each time‐segment: the level, which is the value of the outcome at the beginning of each segment, and the trend, which is the rate of change of the outcome during each segment.

Researchers fit linear mixed effect regression models accordingly to estimate the effect of the intervention on levels and monthly trends of the following outcomes: the proportion of PAC patients per month that accepted LARC and the proportion that accepted any contraceptive method, respectively. Changes in the level represented an abrupt intervention effect, and changes in the trend represented gradual change that occurred over time. Investigators assessed several model specifications to identify the best for mainland Tanzania and Zanzibar hospital data. For both regions, the best models used random intercepts to account for facility‐level correlation in the outcome over time and were fit by maximum likelihood methods. Researchers observed that on the mainland there was no level change in the outcome associated with the transition from intervention to sustainment phase, whereas in Zanzibar there was. Therefore, the model they used for the mainland included only one set of time‐segmentation parameters, while the Zanzibar model included two sets of terms to account for the fixed effects of onset and change in trend associated with the intervention and sustainment phases. Both models adjusted for a vector of covariates on facility type (regional referral or district hospital), district, and year to account for potential sources of unmeasured confounding. Statistical analysis was conducted using R version 4.0.2.

We received ethical approval from the Tanzanian National Institute of Medical Research. The US‐based Western Internal Review Board approved the study protocol. No consent was sought from individual patients because we only used de‐identified facility data routinely collected in registers.

## 3 RESULTS

Tables 1 and 2 illustrate the sociodemographic characteristics of PAC patients for each period for mainland and Zanzibar hospitals. Despite its smaller population, hospitals in Zanzibar received a larger number of PAC patients than those in *PAC‐FP* sites on the mainland. This is because until 2018 with support from *PAC‐FP*, PAC services had not been formally introduced in Zanzibar primary care settings. This contrasts with mainland Tanzania, where non‐governmental organization‐led initiatives supported the scale up of PAC at lower‐level sites starting in the 2000s.

**TABLE 1:** Sociodemographic and PAC service delivery characteristics for pre‐intervention (January 2014 to July 2016), intervention (August 2016 to October 2018) and sustainment (November 2018 to June 2020) phases (mainland Tanzania)^a^

	Time segments (2014–2020)		
	Pre‐intervention	Intervention	Sustainment
Age, y			
<20	308 (12)	281 (12)	150 (13)
20–24	729 (29)	650 (28)	330 (28)
25–29	620 (25)	533 (23)	265 (22)
30–34	433 (17)	420 (18)	214 (18)
35–39	264 (11)	272 (12)	156 (13)
≥40	142 (6)	162 (7)	83 (7)
Missing	5 (<1)	–	2 (<1)
Marital status			
Married/in‐union	2141 (85)	1991 (85)	936 (78)
Unmarried	318 (13)	287 (13)	97 (8)
Missing	42 (2)	40 (2)	167 (14)
Parity			
0	410 (16)	440 (19)	156 (13)
1	417 (17)	401 (17)	213 (18)
2	428 (17)	421 (18)	225 (19)
3	398 (16)	343 (15)	147 (12)
4	314 (13)	216 (9)	114 (9)
≥5	431 (17)	414 (18)	203 (17)
Missing	103 (4)	83 (4)	142 (12)
Mean gestational age	9 weeks	8 weeks	8 weeks
PAC provider type			
Nurse	1038 (42)	964 (41)	513 (43)
Midwife	680 (27)	709 (31)	338 (28)
Physician	780 (31)	641 (28)	347 (29)
Missing	3 (<1)	4 (<1)	2 (<1)
Treatment method			
Manual vacuum aspiration or electric vacuum aspiration	2453 (98)	2223 (96)	1149 (96)
Misoprostol	39 (2)	75 (3)	33 (3)
Sharp curettage	9 (<1)	20 (1)	18 (1)
Missing	–	–	
Pain relief given			
Yes	2500 (100)	2318 (100)	1200 (100)
No	1 (<1)	0 (0)	0 (0)
Missing	–	–	–
FP counseling			
Yes	2496 (100)	2262 (98)	1076 (90)
No	5 (<1)	56 (2)	124 (10)
Missing	–	–	–
Contraceptive uptake			
None	83 (3)	162 (7)	175 (15)
Short‐term method	2140 (86)	928 (40)	615 (51)
LARC	278 (11)	1228 (53)	410 (34)
Missing	–	–	–
Total	2501 (100)	2318 (100)	1200 (100)

Abbreviations**:** FP, family planning; LARC, long‐acting reversible contraception; PAC, postabortion care.

^a^ Values are presented as number (percentage) or as mean.

**TABLE 2:** Sociodemographic and PAC service delivery characteristics for pre‐intervention (January 2014 to July 2016), intervention (August 2016 to October 2018) and sustainment (November 2018 to June 2020) phases (Zanzibar)^a^

	Pre‐intervention	Intervention	Sustainment
Age, y			
<20	164 (7)	331 (7)	262 (6)
20–24	565 (26)	1121 (25)	1156 (26)
25–29	539 (24)	1161 (25)	1161 (26)
30–34	389 (18)	873 (19)	824 (19)
35–39	328 (15)	707 (15)	636 (14)
≥40	177 (8)	332 (7)	336 (8)
Missing	44 (2)	41 (<1)	21 (<1)
Marital status			
Married/in‐union	2067 (94)	4356 (95)	4107 (94)
Unmarried	89 (4)	197 (4)	180 (4)
Missing	50 (2)	13 (<1)	109 (2)
Parity			
0	380 (17)	722 (16)	812 (18)
1	309 (14)	628 (14)	647 (15)
2	298 (14)	643 (14)	663 (15)
3	205 (9)	529 (12)	548 (12)
4	178 (8)	383 (8)	421 (10)
≥5	388 (18)	860 (19)	777 (18)
Missing	448 (20)	801 (17)	528 (12)
Mean gestational age	11 weeks	9 weeks	8 weeks
PAC provider type			
Nurse	475 (22)	2172 (48)	2872 (65)
Midwife	0 (0)	4 (<1)	1524 (35)
Physician	1722 (78)	2385 (52)	0 (0)
Missing	9 (<1)	4 (<1)	–
Treatment method			
Manual vacuum aspiration or electric vacuum aspiration	1378 (63)	4196 (92)	3775 (86)
Misoprostol	731 (33)	250 (5)	507 (12)
Sharp curettage	95 (4)	118 (3)	112 (2)
Missing	2 (<1)	4 (<1)	1 (<1)
Pain relief given			
Yes	2205 (100)	4563 (100)	4395 (100)
No	0	1 (<1)	1 (<1)
Missing	1 (<1)	2 (<1)	–
FP counseling			
Yes	2120 (96)	4462 (98)	4244 (97)
No	38 (2)	101 (2)	152 (3)
Missing	48 (2)	3 (<1)	–
Contraceptive uptake			
None	1979 (90)	2181 (48)	1316 (30)
Short‐term method	203 (9)	1806 (39)	2403 (55)
LARC	23 (1)	577 (13)	677 (15)
Missing	1 (<1)	2 (<1)	–
Total	2206 (100)	4566 (100)	4396 (100)

Abbreviations**:** FP, family planning; LARC, long‐acting reversible contraception; PAC, postabortion care.

^a^ Values are presented as number (percentage) or as mean.

**FIGURE 2:**
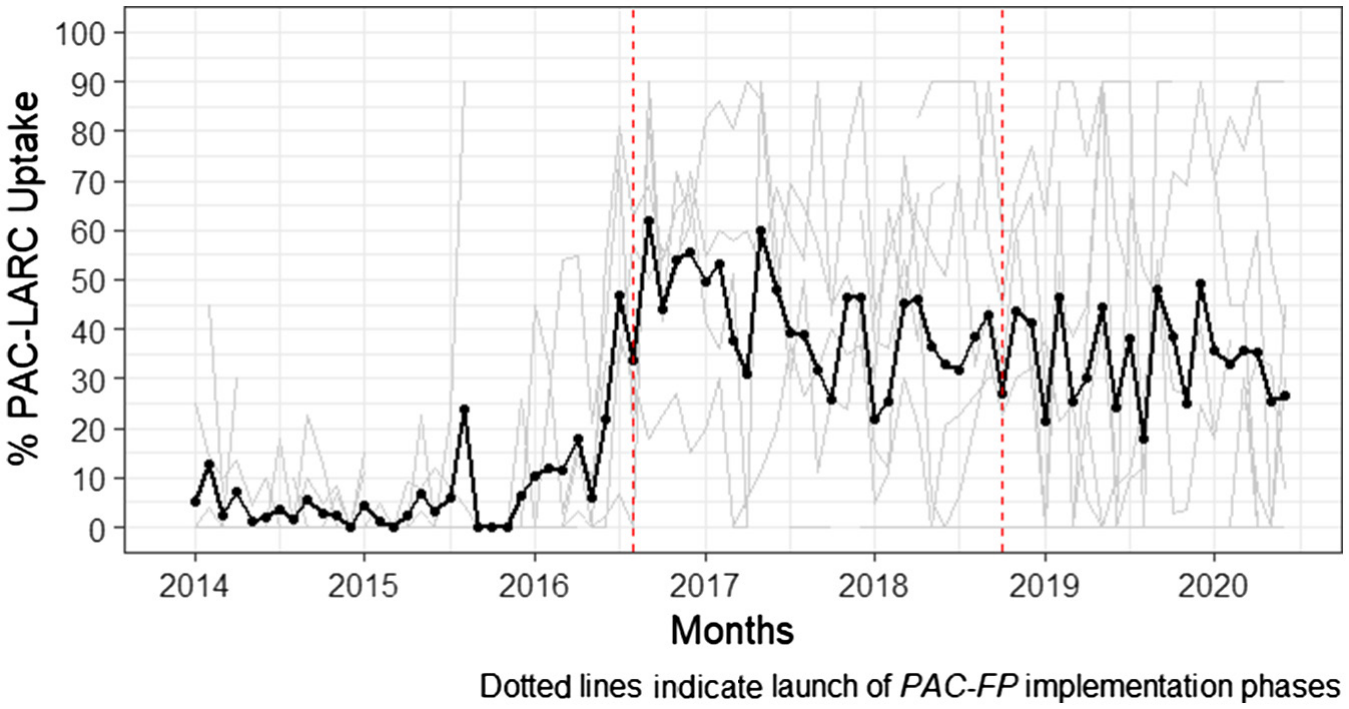
Proportion of PAC patients in mainland hospitals that chose LARC, January 2014 to June 2020

The model fit for the mainland Tanzania interrupted time series reports that during the period before the interventions, postabortion LARC acceptance had started to trend upward. This increase may be a result of *PAC‐FP*’s implementation of steps 1 and 2, during the first half of 2016. The launch of step 3, however, was associated with an immediate average 38 percentage‐point level change in the proportion of PAC patients per facility who accepted LARC before discharge (*P* < 0.001). After the trainings that occurred in Step 3, the QI interventions were associated with a gradual and small decline in postabortion LARC uptake per month, which, on average, trended at approximately 34% during the sustainment period (β = −0.01, *P* < 0.001), over 20 percentage‐points higher than in the first time‐segment (Figure 2). Unlike postabortion LARC trends, uptake of any postabortion contraception was stable throughout the intervention in *PAC‐FP*’s mainland facilities. Our model reports a gradual decline in the average proportion of women accepting postabortion FP per month before the intervention, which continued after the project start (β = −0.01, *P* < 0.001). Nevertheless, as Table 1 attests, levels of this outcome in mainland facilities remained very high throughout the project. During the 2 years that followed the launch of the intervention, approximately 53% of PAC patients accepted LARC, of whom 282 (23%) chose an IUD and 946 (77%) chose an implant; whereas during the sustainment period starting in late 2018—when postabortion LARC uptake declined—only 62 patients (15%) accepted an IUD, whereas 348 (85%) accepted an implant.

**FIGURE 3:**
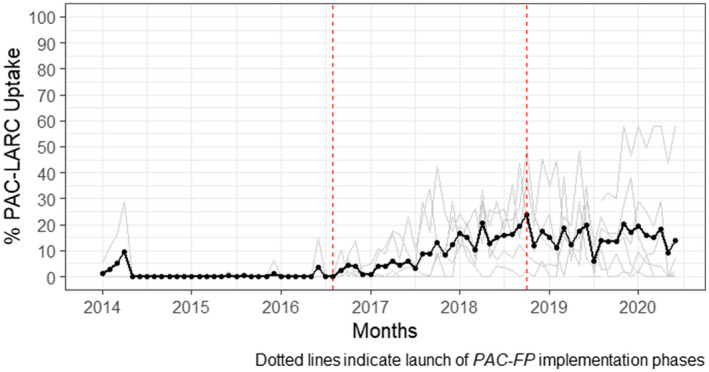
Proportion of PAC patients in Zanzibar hospitals that chose LARC, January 2014 to June 2020

**FIGURE 4:**
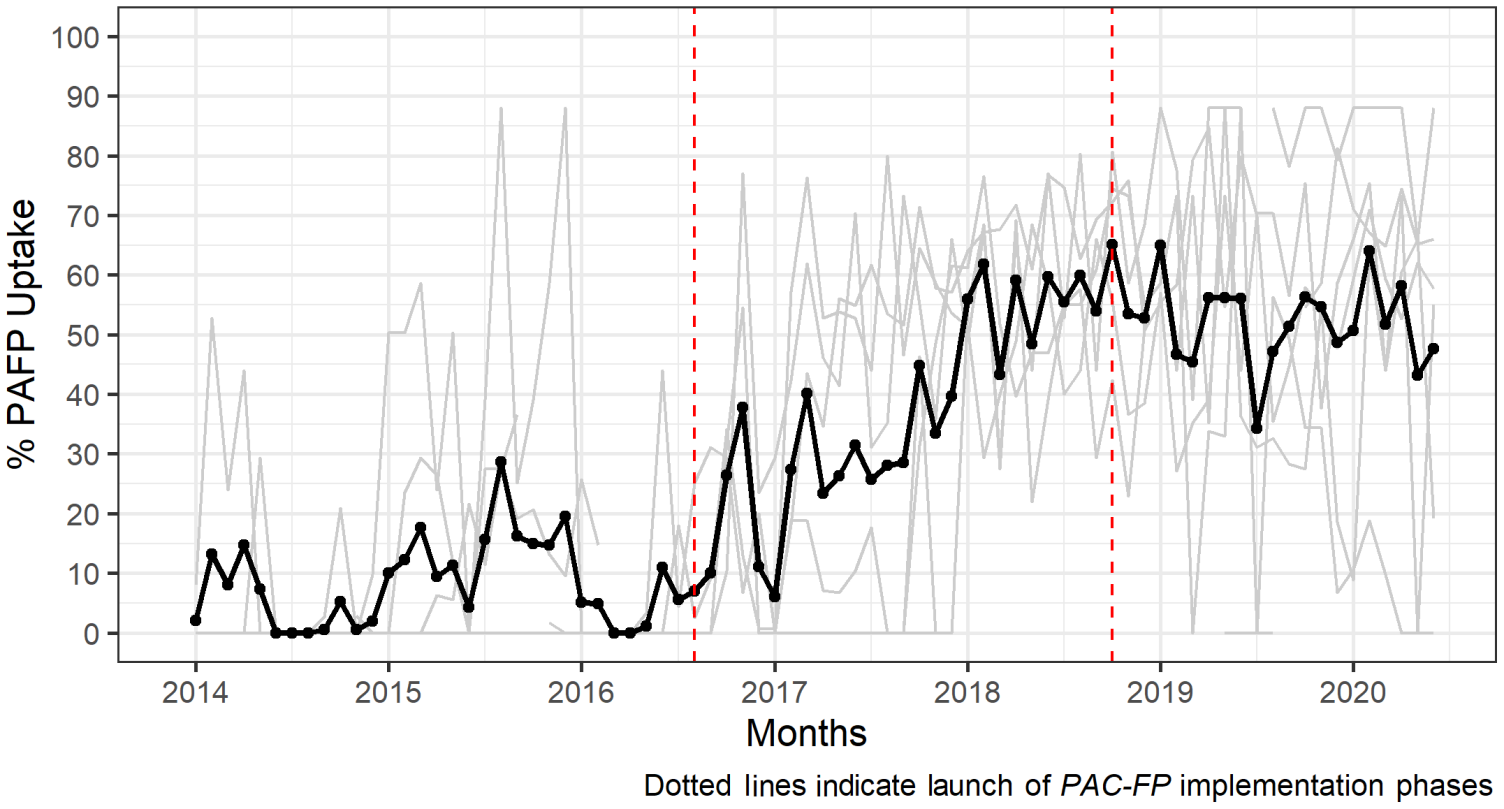
Proportion of PAC patients in Zanzibar hospitals that accepted contraception, January 2014 to June 2020

Figures 3 and 4 illustrate the effect of *PAC‐FP* interventions in Zanzibar. Trends of the postabortion LARC uptake present a contrast to those observed at mainland sites. In Zanzibar, the launch of interventions in mid‐2016 achieved no immediate effect of LARC uptake among PAC patients; however, these interventions were associated with a gradual increase in the monthly rate of change of this outcome (β = 0.01, *P* < 0.001). This resulted in the average proportion of PAC patients to accept LARC in Zanzibar hospitals peaking at approximately 25% in the final quarter of 2018, coinciding with the Zanzibar Ministry of Health’s introduction of clinical guidelines on PAC. The model fit to assess postabortion LARC uptake trends in Zanzibar reports that the period of the guidelines launch was associated with a small decline and then stabilization of this trend through 2018, in contrast to the gradual upward trends observed during the previous time‐segment (β = −0.01, *P* < 0.001).

As with postabortion LARC, there was no immediate level change in uptake of any method among PAC patients; however, the interventions were associated with a much more precipitous increase in the rate of change of the outcome during the second time‐segment (β = 0.02, *P* < 0.001). The analysis also suggests that these increases, after peaking at approximately 65% during the final quarter of 2018, remained stable throughout the sustainment period until the end of the time series (β = −0.02, *P* < 0.001). During the 2‐year period following the launch of interventions in Zanzibar, among the 1806 PAC patients that accepted a short‐term method, 132 (7%) chose a condom, 678 (38%) an oral contraceptive, and 993 (55%) an injectable method, and of the 577 PAC that accepted a LARC, 99 (17%) chose an IUD and 478 (83%) an implant. However, in the sustainment period that started approximately 2 years after the launch, among the 2403 PAC patients that accepted a short‐term method, 107 (4%) chose a condom, 1009 (42%) an oral contraceptive, and 1287 (51%) an injectable method, and of the 677 PAC that accepted a LARC, 161 (23%) chose an IUD and 516 (77%) an implant.

Table 3 presents the results of the linear mixed effects segmented regression analyses.

**TABLE 3:** Results of linear mixed effects segmented regression analysis of uptake of postabortion LARC and any modern contraceptive method for PAC‐FP sites in mainland Tanzania and Zanzibar

	β estimate	95% CI lower bound	95% CI upper bound	*P* value
Model 1: Linear mixed effects segmented regression of postabortion LARC uptake in mainland hospitals				
Month before intervention (2014–2016)^a^	0.01	0.00	0.01	<0.001
Intervention (2016)^b^	0.38	0.28	0.48	<0.001
Month after intervention (2016–2020)^c^	−0.01	−0.01	0.00	<0.001
Model 2: Linear mixed effects segmented regression of postabortion contraceptive uptake in mainland hospitals				
Month before intervention (2014–16)^a^	−0.01	−0.01	0.00	<0.001
Intervention (2016)^b^	0.01	−0.07	0.09	0.754
Month after intervention (2016–2020)^c^	−0.01	−0.01	0.00	<0.001
Model 3: Linear mixed effects segmented regression of postabortion LARC uptake in Zanzibar hospitals				
Month before intervention (2014–2016)^a^	0.00	0.00	0.00	0.632
Intervention (2016)^b^	−0.01	−0.09	0.07	0.850
Month after intervention (2016–2018)^d^	0.01	0.01	0.02	<0.001
Sustainment (2018)^e^	−0.03	−0.11	0.05	0.414
Month after sustainment (2018–2020)^f^	−0.01	−0.02	−0.01	<0.001
Model 4: Linear mixed effects segmented regression of postabortion contraceptive uptake in Zanzibar hospitals				
Month before intervention (2014–2016)^a^	0.00	0.00	0.01	0.662
Intervention (2016)^b^	−0.01	−0.13	0.12	0.933
Month after intervention (2016–2018)^d^	0.02	0.01	0.03	<0.001
Sustainment (2018)^e^	−0.06	−0.19	0.06	0.301
Month after sustainment (2018–2020)^f^	−0.02	−0.03	−0.01	<0.001

Abbreviations**:** LARC, long‐acting reversible contraception; *PAC‐FP*, Postabortion Care Family Planning Project.

^a^ The average month‐to‐month rate of change among hospitals in the outcome from January 2014 to July 2016 (0.01 = 1% per month).

^b^ The immediate level change among hospitals associated with the onset of the intervention in August 2016.

^c^ The difference between the average month‐to‐month rate of change among hospitals in the outcome comparing January 2014 with July 2016 and August 2016 with June 2020.

^d^ The difference between the average month‐to‐month rate of change among hospitals in the outcome comparing January 2014 with July 2016 and August 2016 with October 2018.

^e^ The immediate level change among hospitals associated with the onset of the sustainment phase in November 2018.

^f^ The difference between the average month‐to‐month rate of change among hospitals in the outcome comparing August 2016 with October 2018 and November 2018 with June 2020.

## 4 DISCUSSION

Our study sought to assess changes in the integration of treatment for abortion complications and FP services that were associated with a multi‐year, phased intervention that aimed at strengthening systems and improving the quality of PAC in two distinct regions of Tanzania. In Geita and Mwanza, where PAC service strengthening has long‐standing historical roots, *PAC‐FP* interventions aimed at LARC integration were successful, catalyzing a sharp increase in the provision of IUDs and hormonal implants in the immediate term, and, in the long run, resulting in a more evenly balanced contraceptive method mix accessed by PAC patients that was sustained over time. It should be noted, however, that the incidence of postabortion LARC uptake in mainland hospitals declined after the initial period following the intervention, indicating a temporary diminishing effect of time after training. Nevertheless, trends illustrate that 2 years after the intervention launch and onward, the proportion of PAC patients accepting LARC in these settings plateaued at levels appreciably greater than they had been in the period before the intervention. In Zanzibar, where technical assistance to the PAC program had been scarcer, strengthening FP integration with PAC services gradually also proved effective, most saliently by instigating continuous rises in patients' uptake of modern methods, albeit with more emphasis on short‐acting methods than LARC, which endured throughout the sustainment phase.

The analysis contributes to existing knowledge on this topic. Whereas earlier studies have demonstrated that strategies to adapt the organization of care,^11^, ^12^ promote a more enabling policy environment,^13^ strengthen woman‐centered counseling,^14^ and enhance the uptake of contraception after PAC, *PAC‐FP* has generated new knowledge on the effects of embedding a participatory, teamwork‐based approach to promoting uptake of best practices by customizing systems strengthening and QI approaches to distinct contexts of local health systems at different stages of PAC services development. This study, furthermore, stands out from others on the scale up of PAC that report on trends over shorter periods, and complements existing studies on the institutionalization of PAC in health systems by analyzing the immediate and long‐term effects of embedded implementation strategies years after an external assistance project commenced.^15^, ^16^

It is also important to acknowledge the room for improvement, particularly in Zanzibar. Postabortion LARC utilization remained low throughout the time series and although this study was not designed to ascertain the cause, and whether or not low uptake of LARC reflects patients' true preference, studies conducted in the same hospitals report high patient volumes, overcrowded facilities, time constraints, and inadequate staffing.^17^ Indeed, an objective of *PAC‐FP* through its support of PAC guidelines in Zanzibar was to promote decentralization of PAC from tertiary to primary care settings and thereby help to decongest hospitals. The results shared here suggest that those efforts were too nascent for the results of the complex systems changes they aimed to trigger to appear in this analysis. Earlier studies that compare postabortion FP uptake between the PAC‐FP mainland and Zanzibar sites illustrate demand‐side constraints to the uptake of LARC that are more prominent in the latter.^10^ This underscores the importance of social and behavioral change communication, addressing adverse gender norms, and strengthening primary healthcare systems to accelerate improvements in the Zanzibar PAC program. Although improvements in postabortion LARC uptake lasted in mainland hospitals, performance declined during sustainment. This study could not ascertain the reasons for this, but mainland sites may have experienced challenges in sustaining QI over the long‐term. Additional interventions and research that promote the routinization of QI and analyze adherence to specific aspects of QI strategies over time may help to address this problem. Finally, the onset of the COVID‐19 pandemic in March of 2020 may have affected patient access to facilities and disrupted service provision, resulting in a drop in LARC uptake in mainland hospitals and in postabortion FP uptake in Zanzibar hospitals in the final months of the project.

Nevertheless, the experience reported in this study is encouraging: by customizing conventional systems strengthening and QI approaches to distinct, fragile local health systems and service delivery contexts, it is possible to achieve large and sustainable improvements in the voluntary uptake of FP, including LARC, among postabortion patients.

## AUTHOR CONTRIBUTIONS

CB designed the study, led the data analysis, and wrote the manuscript with input from DG, JK, and KOC. JK supervised and led components of the data collection and coordinated fieldwork. All authors discussed the results and contributed to the final manuscript.

## CONFLICTS OF INTEREST

The authors declare that they have no competing interests.

## Data Availability

Research data are not shared.
